# *MMP‐14* single‐nucleotide polymorphisms are related to steroid‐induced osteonecrosis of the femoral head in the population of northern China

**DOI:** 10.1002/mgg3.519

**Published:** 2018-12-12

**Authors:** Yuxin Qi, Jiaqi Wang, Mingqi Sun, Chao Ma, Tianbo Jin, Yuan Liu, Yuju Cao, Jianzhong Wang

**Affiliations:** ^1^ Inner Mongolia Medical University Hohhot China; ^2^ The 2nd Affiliated Hospital of Inner Mongolia Medical University Hohhot China; ^3^ Zhengzhou TCM Traumatology Hospital Zhengzhou China; ^4^ MOE Key Laboratory of Resource Biology and Modern Biotechnology Northwest University Xi'an China

**Keywords:** association study, *MMP‐14*, single‐nucleotide polymorphisms, steroid‐induced osteonecrosis of the femoral head

## Abstract

**Background:**

Osteonecrosis of the femoral head (ONFH) is a refractory disease which frequently occurs in young and middle‐aged people. Recent studies indicated that MMP‐14 played an important role in the development of chondrocytes, metabolism of osteoblasts as well as fate decision of hypertrophic chondrocytes. The aim of this study was to investigate the association between polymorphisms of *MMP‐14* and steroid‐induced osteonecrosis of the femoral head in the Chinese population.

**Methods:**

We selected 7 SNPs (rs3751488, rs1003349, rs1042703, rs2236302, rs1042704, rs2236303, and rs2236304) on gene *MMP‐14*. Odds ratios (ORs) and 95% confidence intervals (CIs) were estimated using the chi‐squared test, genetic model analysis, haplotype analysis, and stratification analysis.

**Results:**

We discovered that the genotype “G/G” of rs2236302 was associated with ONFH risk in the *MMP‐14* in the codominant model (OR = 8.62, 95% CI = 1.07–69.46, *p* = 0.038) and recessive model (OR = 8.86, 95% CI = 1.10–71.31, *p* = 0.013).

**Conclusions:**

We have confirmed that the susceptive SNPs (rs2236302) of *MMP‐14* from the *MMPs/TIMPs* system exhibit a significant association with increased risk of steroid‐induced ONFH in the population of northern China.

## 1 INTRODUCTION

Osteonecrosis of the femoral head (ONFH) is a refractory disease which frequently occurs in young and middle‐aged people. Widespread use of glucocorticoids is one of the main causes of osteonecrosis of the femoral head. Steroid‐induced ONFH accounts for 24.1% of all femoral head necrosis (Cui et al., 2015). Since this stubborn disease may seriously affect the quality of life for patients, a large number of researchers have kept an eye on the identification of risk factors for ONFH. Recent studies show that genetic polymorphisms of enzymes play essential roles in contributing to individual differences in steroid metabolism, steroid receptors, and transport proteins in patients with steroid‐induced ONFH (He & Li, 2009; Kuribayashi et al., 2008). Genetic factors have been demonstrated to be a strong factor of this disturbance. Many candidate genes have been shown related to ONFH in previous studies (Hadjigeorgiou et al., 2008).

Matrix metalloproteinases (MMPs) are a large class of secretory proteases involved in normal physiological and pathological processes such as embryogenesis, wound healing, angiogenesis, tissue remodeling, tumor invasion, and metastasis (Knäuper & Murphy, 1998). With the development of MMPs research both at home and abroad, the key role of MMPs in bone development, remodeling, and pathological process has attracted much attention.

Studies have shown that MMP‐1 and MMP‐3 are the key enzymes in the formation of bone surface collagen, which can promote bone resorption and achieve the purpose of bone repair (Nakai et al., 2013; Rubin, Sun, Hadjiargyrou, & McLeod, 2010). Though certain MMPs are expressed in bone and cartilage tissue during the normal bone development, MMP‐2, MMP‐9, MMP‐13, MMP‐14, and MMP‐16 play an essential part in skeletal development, as shown by knockout mice models and human genetic diseases (Pagemccaw, Ewald, & Werb, 2007; Paiva & Granjeiro, 2014). Viable transplantation studies show that MMP‐9 may be involved in bone regeneration mediated by stem cells (Mosig et al., 2007). In our previous study, we have demonstrated that *MMP‐8,MMP‐9* single‐nucleotide polymorphisms are related to steroid‐induced ONFH in Chinese population (Du, Jin, et al., 2016; Du, Lin, et al., 2016).

MMP14 (alias membrane type 1 MMP, MT1‐MMP) especially is related to many processes including wound healing, angiogenesis, inflammation, and cancer invasion and metastasis (Albrechtsen et al., 2013; Azar et al., 2010; Moss, Jensen‐Taubman, & Stetler‐Stevenson, 2012). Recent studies indicate that MMP14 play an important role in the development of chondrocytes, metabolism of osteoblasts as well as fate decision of hypertrophic chondrocytes (Chu, Tsang, Zhou, & Cheah, 2017). The purpose of this study was to understand the polymorphism of *MMP14* gene and its tendency to develop steroid‐induced ONFH in Chinese patients. We conducted a case–control study to analyze the relevance between seven single‐nucleotide polymorphisms (SNPs) in *MMP‐14* and the risk of steroid‐induced ONFH in a Chinese Han population.

## 2 MATERIALS AND METHODS

### 2.1 Ethics statement

The use of human tissue and the protocol in this study was abided by the principles of the Declaration of Helsinki and was approved by the Ethical Committee of Zhengzhou Traditional Chinese Medicine Traumatology Hospital. All candidate subjects signed informed consent.

### 2.2 Study population

We recruited a total of 285 patients diagnosed with steroid‐induced ONFH and 308 control subjects were consecutively enrolled from 2014 to 2017 among Han Chinese. Steroid‐induced ONFH was defined as having a history of an average daily steroid dose of ≥16.6 mg, or a high‐dose steroid impulsion therapy lasting more than 1 week (Koo et al., 2002; Zhang et al., 2014). All the subjects were treated by the Affiliated Zhengzhou Traditional Chinese Medicine Traumatology Hospital. All cases were verified, and patients were recruited without age, sex, or disease stage restriction. Moreover, patients did not receive systemic inflammatory treatment including drug control treatment before the blood samples used in this study were obtained.

A number of 308 healthy unrelated individuals were recruited randomly as sample, and the participants were Han Chinese living in Zhengzhou city and nearby. All of the chosen subjects were from the Zhengzhou Traditional Chinese Medicine Traumatology Hospital. To reduce the potential environmental and therapeutic factors impacting the variation of complex human diseases, we performed detailed recruitment and set exclusion criteria to exclude subjects with diseases related to genetic susceptibility, such as tumor.

### 2.3 SNP selection and genotyping

We selected seven SNPs for investigation in this study. We prioritized SNPs to be studied considering: (a) previous reports of expression in diseased tissues, (b) previous reports of association with ONFH, (c) substrates as recognized molecules in diseased tissues.

Within selected SNPs, seven polymorphisms were selected based on published reports and/or their locations in the genes, based on their likelihood to have functional consequences (i.e., located in the promoters, exons or near exon/intron boundaries), or if considered tag SNPs as surrogates for the linkage disequilibrium blocks surrounding the candidate gene. We used information from the NCBI dbSNP (http://www.ncbi.nlm.nih.gov/snp).

A total of seven tSNPs in the *MMP14* gene were selected for further genotyping. The phenol–chloroform extraction method was performed to extract genomic DNA from whole blood (Holmbeck et al., 2005). DNA concentration was measured by spectrometry (DU530 UV/VIS spectrophotometer, Beckman Instruments, Fullerton, CA, USA). Sequenom MassARRAY Assay Design 3.0 software was used to design multiplexed SNP MassEXTEND assay, and SNP genotyping was performed utilizing the Sequenom MassARRAY RS1000 recommended by the manufacturer (Gabriel, Ziaugra, & Tabbaa, 2009). Data management and analyses were performed using the Sequenom Typer 4.0 software as previously described (Gabriel et al., 2009; Thomas et al., 2007).

## 3 RESULTS

A total of 285 cases and 308 controls were included in this study. The demographics in steroid‐induced ONFH cases and control are shown in Table 1. We selected seven SNPs (rs3751488, rs1003349, rs1042703, rs2236302, rs1042704, rs2236303, and rs2236304) on gene *MMP‐14*. The basic SNP information of all subjects investigated was listed in Table 2, including position, alleles, the minor allele frequency (MAF), odds ratios (ORs), 95% confidence intervals (95% CIs), and the *p*‐values of alleles evaluated by chi‐squared test. All selected SNPs are inconsistent with the Hardy–Weinberg equilibrium (HWE) (*p* > 0.05). All primers used for genotyping in this study are listed in Table 3.

**Table 1:** Characteristics of cases and controls in this study

Variable(s)	Case (*n *=* *285)	Control (*n* = 308)	*p* value
Sex *N* (%)			
Male	173	197	<0.001^a^
Female	113	112	
Age, year (mean ± *SD*)	41.83 ± 13.14	48.75 ± 8.42	<0.001^b^

^a^Two‐sided chi‐squared test. ^b^Independent samples *t* test.

**Table 2:** Allele frequencies in cases and controls and odds ratio estimates for steroid‐induced ONFH

SNP ID	Gene	Position	Alleles	MAF		*p* value^a^ for HWE	Ors	95% CI		*p* b
			A/B	Case	Control					
rs3751488	MMP14	14q11.2	A/G	0.291	0.301	1	0.9523	0.742	1.223	0.701489
rs1003349	MMP14	14q11.2	T/G	0.474	0.441	0.2987	1.1396	0.906	1.433	0.264037
rs1042703	MMP14	14q11.2	C/T	0.005	0.008	1	0.6443	0.153	2.708	0.802734
rs2236302	MMP14	14q11.2	G/C	0.129	0.118	0.09696	1.1094	0.785	1.567	0.55576
rs1042704	MMP14	14q11.2	A/G	0.026	0.031	1	0.8462	0.426	1.682	0.633195
rs2236303	MMP14	14q11.2	T/C	0.505	0.468	0.4232	1.163	0.926	1.461	0.193778
rs2236304	MMP14	14q11.2	G/C	0.467	0.452	0.4175	1.0595	0.842	1.333	0.621031

*Notes*

Bonferroni's multiple adjustment was applied to the level of significance, which was set at *p* ≤ 0.00104 (0.05/48). HWE: Hardy–Weinberg equilibrium; MAF: minor allele frequency; OR: odds ratio, SNP: single‐nucleotide polymorphism; 95% CI, 95% confidence interval.

^a^p was calculated by exact test. ^b^p was calculated by Pearson chi‐squared test.

**Table 3:** Primers used for this study

SNP_ID	1st‐PCRP	2nd‐PCRP	UEP_SEQ
rs3751488	ACGTTGGATGTGTCCCCTTTTCACGTTCAC	ACGTTGGATGAAATTTACCTCCTTTGCAG	tgCCTTTGCAGGCCATA
rs1003349	ACGTTGGATGCTGCACCACAAAAAGGCAAC	ACGTTGGATGGACGTGGTTGTTTTAGCCTG	acttAATCCAATTACAACCAAGAA
rs1042703	ACGTTGGATGCTGAAGCTGCTGCTTTGGG	ACGTTGGATGTGGTCTCGGACCATGTCTC	CCCGCCCCAAGACCC
rs2236302	ACGTTGGATGGCTCGAGCATTCCAGTGAC	ACGTTGGATGGCACAAAATTCTCCGTGTCC	GTGTCCATCCACTGGTAAAA
rs1042704	ACGTTGGATGTTTACCAGTGGATGGACACG	ACGTTGGATGCATAAAGTTGCTGGATGCCC	CCCGGCGGTCATCAT
rs2236303	ACGTTGGATGAAAAGTGGAGCTGATAGAGG	ACGTTGGATGTCCTGGTCTACGCATTTCCC	ATGCCTTGCAGTCTC
rs2236304	ACGTTGGATGGGCATTTCAGACTTAGGAGG	ACGTTGGATGAAGGGTATTGTCTGCCCATC	CTGCCCATCTGTCTGT

We proposed a hypothesis that each minor allele was compared with the corresponding wild‐type allele. We established five different genetic models that assessed the association between SNPs and steroid‐induced ONFH risk by using unconditional logistic regression. As a result, we discovered that the genotype “G/G” of rs2236302 (HGVS: CM000676.2:g.22843345C>A) was associated with steroid‐induced ONFH risk in the *MMP‐14* in the codominant model (OR = 8.62, 95% CI = 1.07–69.46, *p* = 0.038) and recessive model (OR = 8.86, 95% CI = 1.10–71.31, *p* = 0.013).As is shown in Table 4, a rigorous Bonferroni correction analysis was applied so as to reduce the potential of spurious findings due to multiple testing. It was a pity that the difference was no longer significant after Bonferroni correction.

**Table 4:** Genotypic model analysis of relationship between SNPs and steroid‐induced ONFH risk

SNP ID	Model	Genotype	Group = control	Group = hormone	Without adjustment		With adjustment	
					OR (95% CI)	*p*‐value^a^	OR (95% CI)	*p*‐value^a^
rs2236302	Codominant	C/C	237 (76.7%)	220 (76.9%)	1	0.027^b^	1	0.038^b^
		G/C	71 (23%)	58 (20.3%)	0.88 (0.59–1.30)		0.89 (0.59–1.34)	
		G/G	1 (0.3%)	8 (2.8%)	8.62 (1.07–69.46)		8.37 (1.00–69.71)	
	Dominant	C/C	237 (76.7%)	220 (76.9%)	1	0.95	1	0.98
		G/C‐G/G	72 (23.3%)	66 (23.1%)	0.99 (0.67–1.45)		0.99 (0.67–1.48)	
	Recessive	C/C‐G/C	308 (99.7%)	278 (97.2%)	1	0.0089^b^	1	0.013^b^
		G/G	1 (0.3%)	8 (2.8%)	8.86 (1.10–71.31)		8.60 (1.03–71.51)	
	Overdominant	C/C‐G/G	238 (77%)	228 (79.7%)	1	0.42	1	0.46
		G/C	71 (23%)	58 (20.3%)	0.85 (0.58–1.26)		0.86 (0.57–1.29)	
	Log‐additive	—	—	—	1.11 (0.79–1.57)	0.56	1.12 (0.78–1.60)	0.55

Bonferroni's multiple adjustment was applied to the level of significance, which was set at *p* ≤ 0.00104 (0.05/48).

a *p* values were calculated by Wald test by unconditional logistic regression adjusted for age and gender.

b *p* ≤ 0.05.

**Figure 1:**
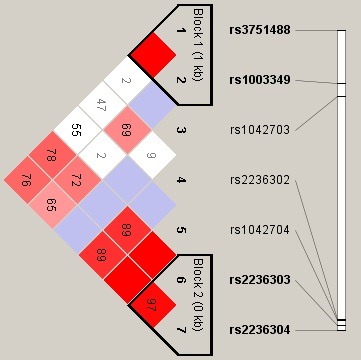
We used the parameters D′ to analyze the linkage disequilibrium (LD) of the SNPs on *MMP‐14*. Significant LD is indicated by bright red standard colors

We use linkage disequilibrium (LD) and haplotype analyses to characterize the SNPs in *MMP‐14*. We calculated LD between seven SNPs, and the haplotype structure of the *MMP‐14* gene was analyzed (*r*²and D′). Block 1 (rs3751488 and rs1003349) and Block 2 (rs2236303 and rs2236304) of *MMP14* SNPs (Figure 1) were found by haplotype analysis. We found that the “TC” haplotype was associated with a significantly increased steroid‐induced ONFH risk after adjustment for age and gender (OR = 2.43, 95% CI = 1.20–4.92, *p* = 0.014). The results are shown in Table 5 and Figure 1.

**Table 5:** The haplotype frequencies of *MMP14* polymorphisms and their association with the risk of steroid‐induced ONFH

Haplotype	rs2236303	rs2236304	Freq	Without adjustment		With adjustment	
				OR (95% CI)	*p* *	OR (95% CI)	*p* **
1	C	C	0.509	1	—	1	—
2	T	G	0.45	1.12 (0.89–1.42)	0.34	1.09 (0.85–1.40)	0.48
3	T	C	0.036	1.92 (1.00–3.70)	0.05	2.43 (1.20–4.92)	0.014***

*p values were calculated by unconditional logistic regression. **p values were calculated by unconditional logistic regression adjusted for age and gender. ***p < 0.05 indicates statistical significance.

## 4 DISCUSSION

Widespread use of hormones in Chinese populations is the most important causes of steroid‐induced ONFH. However, the association between MMP/TIMP gene polymorphisms and steroid‐induced ONFH is rarely reported in Chinese populations. We have reported for the first time the association between *MMP‐14* gene polymorphisms and steroid‐induced ONFH. We researched the associations between seven SNPs in the *MMP‐14* gene and the risk of steroid‐induced ONFH. In this case–control study, we confirmed for the first time that *MMP‐14* gene polymorphism (rs2236302) was associated with a risk of steroid‐induced ONFH. Because the ONFH is a complex disease and its genetic pattern is unclear, different genetic models have been hypothesized. We can also observed that a risk effect for the genotype “G/G” of rs2236302 was associated with ONFH risk in the *MMP‐14* in the codominant model (OR = 8.62, 95% CI = 1.07–69.46, *p* = 0.038) and recessive model (OR = 8.86, 95% CI = 1.10–71.31, *p* = 0.013).

Bone resorption and its formation determine the destruction and repair of bone tissue, which are the basis of bone metabolism. Matrix metalloproteinases (MMPs) which is a group of zinc‐dependent proteolytic enzymes are expressed by stromal cells, infiltrating inflammatory cells, osteoclasts, and cancer cells. *MMP* *‐14*, one of the most important members of *MMPs*, has been implicated in various physiopathological processes for its extracellular matrix degrading and accelerating angiogenesis (Sato et al., 1994; Yoshifumi, 2015).

The high expression of *MMP‐14* in synovial tissue of RA (Rheumatoid arthritis) may promote synovial hyperplasia, pannus formation, and angiogenesis in RA progression. Therefore, we hypothesized that MMP14 also played an important role in the pathogenesis of steroid‐induced osteonecrosis. Overexpression of MMP14 increases the relative risk of steroid‐induced ONFH. The immediate cause of steroid‐induced ONFH might not be caused by polymorphism, but it could be a genetic marker which is in linkage disequilibrium with a certain disease predisposing a locus nearby. It is well known that the SNP occurring in *MMP* gene promoters can affect the expression of MMPs (Zhu, Odeberg, Hamsten, & Eriksson, 2006).

At present, the relationship between *MMP‐14* single‐nucleotide polymorphisms and steroid‐induced ONFH has not been reported. However, it has been studied in some researches that MMP‐14 is involved in the osteocytogenesis. The development and/or maintenance of osteocyte processes and their canaliculi are related to matrix degradation which needs the activity of MMP‐14 (Holmbeck et al., 2005). The appearance of the osteocyte processes occurs in the early stage in the transition of osteoblasts to osteocytes (Palumbo, Palazzini, Zaffe, & Marotti, 1990).

In some studies, the association between *MMP14* gene polymorphisms and other diseases, such as intervertebral disc degeneration, has also been reported (Zhang et al., 2015). About the studies of rs2236302 on *MMP‐14* gene, we found that it has been done in some relevant researches among Endocrine Pancreatic Cancer, squamous cell neoplasia of uterine cervix, and early onset of esophageal adenocarcinoma (Campa et al., 2017; Tee et al., 2012; Wu et al., 2011). In especial, it has been shown to be significantly associated with the early onset of esophageal adenocarcinoma. It is not completely determined whether the rs2236302 polymorphisms of *MMP‐14* can influence the susceptibility or severity in patients with steroid‐induced ONFH. Therefore, it has been hypothesized that the genetic variations in *MMP‐14* can influence the susceptibility to ONFH. In our study, we only found that the SNPs of rs2236302 are associated with a risk of steroid‐induced ONFH. As far as we know, we are the first to report the relation between *MMP‐14* polymorphisms rs2236302 and steroid‐induced ONFH risk, but the conclusion identified should be proved in further studies.

There are important discoveries revealed in our study, but some limitations of this study should be considered when interpreting these results. First of all, our study does not include an analysis of biological functions, which will be crucial for elucidating the role of *MMP‐14* in steroid‐induced ONFH. Secondly, the participants in our study were all Han Chinese individuals recruited from the Zhengzhou Traditional Chinese Medicine Traumatology Hospital, which might involve a selection bias. Thirdly, we also performed Bonferroni correction analysis and it was a pity that the association was no longer statistically significant after Bonferroni correction.

To sum up,we have confirmed for the first time that a susceptive SNPs (rs2236302) of *MMP‐14* from the MMPs/TIMPs system exhibit a significant association with increased risk of steroid‐induced ONFH in the population of northern China. Further functional studies and larger population‐based studies are needed to confirm our results.

## CONFLICT OF INTEREST

The authors declare that there are no conflict of interests.

## References

[mgg3519-bib-0001] Albrechtsen, R. , Kveiborg, M. , Stautz, D. , Vikeså, J. , Noer, J. B. , Kotzsh, A. , … Fröhlich, C. (2013). ADAM12 redistributes and activates MMP‐14, resulting in gelatin degradation, reduced apoptosis and increased tumor growth. Journal of Cell Science, 126, 4707–4720. 10.1242/jcs.129510 24006261

[mgg3519-bib-0002] Azar, D. T. , Casanova, F. H. , Mimura, T. , Jain, S. , Zhou, Z. , Han, K. Y. , & Chang, J. H. (2010). Corneal epithelial MT1‐MMP inhibits vascular endothelial cell proliferation and migration. Cornea, 29, 321–330. 10.1097/ICO.0b013e3181b1165d 20118785 PMC2828536

[mgg3519-bib-0003] Campa, D. , Obazee, O. , Pastore, M. , Panzuto, F. , Liço, V. , Greenhalf, W. , … Canzian, F. (2017). Lack of association for reported endocrine pancreatic cancer risk loci in the PANDoRA Consortium. Cancer Epidemiology Biomarkers and Prevention, 26, 1349 10.1158/1055-9965.EPI-17-0075 28765340

[mgg3519-bib-0004] Chu, T. , Tsang, K. , Zhou, Z. , & Cheah, K. S. E. . (2017). MMP14 and regulation of the hypertrophic chondrocyte to osteoblast lineage.

[mgg3519-bib-0005] Cui, L. , Zhuang, Q. , Lin, J. , Jin, J. , Zhang, K. , Cao, L. , … Weng, X. (2015). Multicentric epidemiologic study on six thousand three hundred and ninety five cases of femoral head osteonecrosis in China. International Orthopaedics, 40, 1–10. 26660727 10.1007/s00264-015-3061-7

[mgg3519-bib-0006] Du, J. , Jin, T. , Cao, Y. , Chen, J. , Guo, Y. , Sun, M. , & Wang, J. (2016). Association between genetic polymorphisms of MMP8 and the risk of steroid‐induced osteonecrosis of the femoral head in the population of northern China. Medicine, 95, e4794 10.1097/MD.0000000000004794 27631232 PMC5402575

[mgg3519-bib-0007] Du, J. , Liu, W. , Jin, T. , Zhao, Z. , Bai, R. , Xue, H. , … Wang, J. (2016). A single‐nucleotide polymorphism inMMP9is associated with decreased risk of steroid‐induced osteonecrosis of the femoral head. Oncotarget, 7, 68434–68441. 27637086 10.18632/oncotarget.12034 PMC5356565

[mgg3519-bib-0008] Gabriel, S. , Ziaugra, L. , & Tabbaa, D. (2009). SNP genotyping using the Sequenom MassARRAY iPLEX platform (pp. 2.12.11–12.12.16). Hoboken, NJ: John Wiley & Sons Inc 10.1002/0471142905.hg0212s60 19170031

[mgg3519-bib-0009] Hadjigeorgiou, G. , Dardiotis, E. , Dardioti, M. , Karantanas, A. , Dimitroulias, A. , & Malizos, K. (2008). Genetic association studies in osteonecrosis of the femoral head: Mini review of the literature. Skeletal Radiology, 37, 1. 17962936 10.1007/s00256-007-0395-2

[mgg3519-bib-0010] He, W. , & Li, K (2009). Incidence of genetic polymorphisms involved in lipid metabolism among Chinese patients with osteonecrosis of the femoral head. Acta Orthopaedica, 80, 325 10.3109/17453670903025378 19626470 PMC2823219

[mgg3519-bib-0011] Holmbeck, K. , Bianco, P. , Pidoux, I. , Inoue, S. , Billinghurst, R. C. , Wu, W. , & Poole, A. R. (2005). The metalloproteinase MT1‐MMP is required for normal development and maintenance of osteocyte processes in bone. Journal of Cell Science, 118, 147 10.1242/jcs.01581 15601659

[mgg3519-bib-0012] Knäuper, V. , & Murphy, G. (1998). Membrane‐type matrix metalloproteinases and cell surface‐associated activation cascades for matrix metalloproteinases. Matrix Metalloproteinases, 1998, 199–218. 10.1016/B978-012545090-4/50009-4

[mgg3519-bib-0013] Koo, K. H. , Kim, R. , Kim, Y. S. , Ahn, I. O. , Cho, S. H. , Song, H. R. , … Wang, G. J. (2002). Risk period for developing osteonecrosis of the femoral head in patients on steroid treatment. Clinical Rheumatology, 21, 299–303. 10.1007/s100670200078 12189457

[mgg3519-bib-0014] Kuribayashi, M. , Fujioka, M. , Takahashi, K. A. , Arai, Y. , Hirata, T. , Nakajima, S. , … Kondo, K. (2008). Combination analysis of three polymorphisms for predicting the risk for steroid‐induced osteonecrosis of the femoral head. Journal of Orthopaedic Science, 13, 297–303. 10.1007/s00776-008-1244-4 18696186

[mgg3519-bib-0015] Mosig, R. A. , Dowling, O. , DiFeo, A. , Ramirez, M. C. , Parker, I. C. , Abe, E. , … Martignetti, J. A. (2007). Loss of MMP‐2 disrupts skeletal and craniofacial development and results in decreased bone mineralization, joint erosion and defects in osteoblast and osteoclast growth. Human Molecular Genetics, 16, 1113–1123. 10.1093/hmg/ddm060 17400654 PMC2576517

[mgg3519-bib-0016] Moss, L. A. S. , Jensen‐Taubman, S. , & Stetler‐Stevenson, WG (2012). Matrix metalloproteinases: Changing roles in tumor progression and metastasis. American Journal of Pathology, 181, 1895–1899. 10.1016/j.ajpath.2012.08.044 23063657 PMC3506216

[mgg3519-bib-0017] Nakai, K. , Kawato, T. , Morita, T. , Iinuma, T. , Kamio, N. , Zhao, N. , & Maeno, M. (2013). Angiotensin II induces the production of MMP‐3 and MMP‐13 through the MAPK signaling pathways via the AT(1) receptor in osteoblasts. Biochimie, 95, 922–933. 10.1016/j.biochi.2012.12.016 23277113

[mgg3519-bib-0018] Pagemccaw, A. , Ewald, A. J. , & Werb, Z (2007). Matrix metalloproteinases and the regulation of tissue remodelling. Nature Reviews Molecular Cell Biology, 8, 221 10.1038/nrm2125 17318226 PMC2760082

[mgg3519-bib-0019] Paiva, K. B. S. , & Granjeiro, JM (2014). Bone tissue remodeling and development: Focus on matrix metalloproteinase functions. Archives of Biochemistry & Biophysics, 561, 74 10.1016/j.abb.2014.07.034 25157440

[mgg3519-bib-0020] Palumbo, C. , Palazzini, S. , Zaffe, D. , & Marotti, G. (1990). Osteocyte differentiation in the tibia of newborn rabbit: An ultrastructural study of the formation of cytoplasmic processes. Cells Tissues Organs, 137, 350 10.1159/000146907 2368590

[mgg3519-bib-0021] Rubin, C. , Sun, Y. Q. , Hadjiargyrou, M. , & McLeod, K. (2010). Increased expression of matrix metalloproteinase‐1 in osteocytes precedes bone resorption as stimulated by disuse: Evidence for autoregulation of the cell's mechanical environment? Journal of Orthopaedic Research, 17, 354–361. 10.1002/jor.1100170309 10376723

[mgg3519-bib-0022] Sato, H. , Takino, T. , Okada, Y. , Cao, J. , Shinagawa, A. , Yamamoto, E. , & Seiki, M. (1994). A matrix metalloproteinase expressed on the surface of invasive tumour cells. Nature, 370, 61–65. 10.1038/370061a0 8015608

[mgg3519-bib-0023] Tee, Y. T. , Liu, Y. F. , Chang, J. T. , Yang, S. F. , Chen, S. C. , Han, C. P. , & Liao, C. L. (2012). Single‐nucleotide polymorphisms and haplotypes of membrane type 1‐matrix metalloproteinase in susceptibility and clinical significance of squamous cell neoplasia of uterine cervix in Taiwan women. Reproductive Sciences, 19, 932–938. 10.1177/1933719112438445 22527983

[mgg3519-bib-0024] Thomas, R. K. , Baker, A. C. , Debiasi, R. M. , Winckler, W. , Laframboise, T. , Lin, W. M. , … Garraway, L. A. (2007). High‐throughput oncogene mutation profiling in human cancer. Nature Genetics, 39, 347–351. 10.1038/ng1975 17293865

[mgg3519-bib-0025] Wu, I. C. , Zhao, Y. , Zhai, R. , Liu, G. , Ter‐Minassian, M. , Asomaning, K. , … Christiani, D. C. (2011). Association between polymorphisms in cancer‐related genes and early onset of esophageal adenocarcinoma. Neoplasia, 13, 386–392. 10.1593/neo.101722 21472143 PMC3071087

[mgg3519-bib-0026] Yoshifumi, I. (2015). Membrane‐type matrix metalloproteinases: Their functions and regulations. Matrix Biology, 44–46, 207–223. 10.1016/j.matbio.2015.03.004 25794647

[mgg3519-bib-0027] Zhang, Y. , Kong, X. , Wang, R. , Li, S. , Niu, Y. , Zhu, L. , … Lin, N. (2014). Genetic association of the P‐glycoprotein gene ABCB1 polymorphisms with the risk for steroid‐induced osteonecrosis of the femoral head in Chinese population. Molecular Biology Reports, 41, 3135–3146. 10.1007/s11033-014-3173-y 24469730

[mgg3519-bib-0028] Zhang, J. , Sun, X. , Liu, J. , Liu, J. , Shen, B. , & Nie, L. (2015). The role of matrix metalloproteinase 14 polymorphisms in susceptibility to intervertebral disc degeneration in the Chinese Han population. Archives of Medical Science, 11, 801–806. 10.5114/aoms.2015.53301 26322093 PMC4548033

[mgg3519-bib-0029] Zhu, C. , Odeberg, J. , Hamsten, A. , & Eriksson, P. (2006). Allele‐specific MMP‐3 transcription under in vivo conditions. Biochemical and Biophysical Research Communications, 348, 1150–1156. 10.1016/j.bbrc.2006.07.174 16904077

